# Melanin and Neuromelanin in Humans: Insights Across Health, Aging, Diseases, and Unexpected Aspects of Fungal Melanogenesis

**DOI:** 10.3390/biom16010061

**Published:** 2025-12-30

**Authors:** Kathleen Hatch, Erin K. Murphy, Radamés J. B. Cordero, Diego Iacono

**Affiliations:** 1 DoW/USU Brain Tissue Repository & Neuropathology Program, Uniformed Services University (USU), Bethesda, MD 20814, USA; kathleen.hatch.ctr@usuhs.edu (K.H.); erin.murphy.ctr@usuhs.edu (E.K.M.); 2 Department of Pathology, F. Edward Hébert School of Medicine, Uniformed Services University (USU), Bethesda, MD 20814, USA; 3 The Henry M. Jackson Foundation for the Advancement of Military Medicine (HJF) Inc., Bethesda, MD 20817, USA; 4 Department of Molecular Microbiology and Immunology, Johns Hopkins Bloomberg School of Public Health, Baltimore, MD 21205, USA; rcorder4@jhu.edu; 5 Department of Neurology, F. Edward Hébert School of Medicine, Uniformed Services University (USU), Bethesda, MD 20814, USA; 6 Neuroscience Program, Department of Anatomy, Physiology & Genetics, Uniformed Services University (USU), Bethesda, MD 20814, USA

**Keywords:** melanin, neuromelanin, substantia nigra, fungi, radiation, radiation absorption, neurodegeneration, neuroprotection, radiosynthesis

## Abstract

Melanin pigments are ubiquitous biopolymers across diverse life forms and play multifaceted roles in cellular defense and environmental adaptation. The specialized neuromelanin in human brains accumulates mainly within catecholaminergic neurons of the substantia nigra and locus coeruleus, serving as a crucial modulator of brain homeostasis, metal detoxification, and oxidative stress responses. The intricate processes of human melanogenesis, encompassing both cutaneous and neuronal forms, are governed by complex genetic networks. Concurrently, melanin in fungi (synthesized through distinct genetic pathways) confers remarkable resistance to environmental stressors, including ionizing radiation. Recent advancements in omics technologies—including transcriptomics, proteomics, metabolomics, and epigenomics—have profoundly enhanced our understanding of neuromelanin’s molecular environment in health, aging, and neurodegenerative conditions such as Parkinson’s disease (PD), Alzheimer’s disease (AD), and other neurological disorders. This article reviews the genetic underpinnings of human melanogenesis and fungal melanogenesis, explores the convergent and divergent evolutionary pressures driving their functions, and synthesizes the rapidly accumulating omics data to elucidate neuromelanin’s critical, and often dual, role in human brain pathology. Moreover, we discuss the intriguing parallels between neuromelanin and fungal melanin, highlighting radioprotection and its potential implications for neuroprotection and astrobiology, with a special emphasis on the need to investigate neuromelanin’s potential for radioprotection in light of fungal melanin’s remarkable protective properties.

## 1. Introduction

Melanin, derived from the Greek word *melas*, meaning “black” or “dark”, represents a diverse group of multifunctional biopolymers characterized by dark pigmentation and remarkable chemical stability. These ubiquitous pigments are found across all kingdoms of life, from bacteria and fungi to plants and animals, where they perform a myriad of functions including photoprotection, thermoregulation, structural reinforcement, and defense against pathogens and environmental toxins [1]. In mammals, melanin is primarily responsible for the coloration of skin, hair, and eyes, and is produced within specialized cells called melanocytes. Beyond its well-known cosmetic roles, melanin also plays critical biological roles, such as scavenging free radicals, chelating metal ions, and modulating immune responses [2]. Moreover, a particularly fascinating and species-specific form of melanin is neuromelanin, found predominantly in the human brain. We focus on neuromelanin as an age-dependent metal–lipid–catechol redox buffer and compile omics data to explain when and why this buffer fails in disease. By comparing neuromelanin with fungal melanins, we propose a set of testable hypotheses and experimental paradigms that exploit fungal systems to probe melanin chemistry and function at a resolution difficult to achieve in human tissue. This is a stimulating concept, one which we feel strongly is necessary for the future of understanding the full role and potential of melanin (and neuromelanin) in health and disease.

Neuromelanin is concentrated within catecholaminergic neurons of the substantia nigra pars compacta, a region critical for motor control, the locus coeruleus, which modulates arousal, attention, and stress responses, the dorsal motor nucleus of the vagus nerve, which mediates parasympathetic motor control of the heart, lungs and digestive tract, and neurons of the cerebellum involved in spatial and social memory as well as aspects of executive function and cognitive flexibility [3,4,5]. Unlike the melanin found in the skin, neuromelanin accumulates progressively with age, appearing as dark, granular cytoplasmic inclusions. Its unique localization and age-dependent accumulation suggest a specialized role in the neurobiology of humans and higher primates, a role that becomes evident in neurodegenerative disorders like Parkinson’s disease (PD), where the degeneration of neuromelanin-containing dopaminergic neurons in the substantia nigra pars compacta is a pathological hallmark [6].

The study of melanin, and particularly neuromelanin, has been revolutionized by the advent of “omics” technologies—genomics, transcriptomics, proteomics, metabolomics, and epigenomics. These high-throughput approaches allow for a systemic investigation of the molecular landscape associated with melanin presence and function, both in healthy states and pathological conditions. By providing comprehensive molecular snapshots, omics data have begun to unravel the intricate interplay between genetic predispositions, environmental factors, and the complex biochemistry of melanin, revealing its double-edged nature as both a neuroprotectant and, under certain circumstances, a contributor to neurotoxicity [7].

Research on neuromelanin is more crucial now than ever due to a growing understanding of its complex and seemingly paradoxical role in brain health and neurodegenerative diseases, particularly PD. In fact, for a long time, neuromelanin was thought to be a simple, inert byproduct of dopamine metabolism with a primarily protective function. It was known to accumulate with age, acting like a “sponge” to sequester and neutralize toxic substances like heavy metals (e.g., iron, lead, mercury) and other harmful compounds. However, recent studies have revealed a more complex picture. For example, neuromelanin helps protect neurons by binding to toxic compounds and metal ions, thereby preventing them from causing damage. This is particularly important for dopamine-producing neurons in the substantia nigra, which are especially vulnerable to oxidative stress. Curiously, the paradox is that when neurons start to degenerate, they release their neuromelanin stores into the extracellular space. This released neuromelanin, now with its accumulated load of toxins, can trigger a chronic neuroinflammatory response by activating microglia (the brain’s immune cells). This inflammation creates a “vicious cycle” that further damages and kills neurons, accelerating the progression of diseases like PD. Researchers are currently investigating at what point neuromelanin accumulation crosses a “pathogenic threshold” and goes from being protective to being a source of harm. Among the strongest reasons for the urgency in neuromelanin research is its direct link to PD. The hallmark of PD is the selective degeneration of neuromelanin-containing neurons, particularly in the substantia nigra. By studying neuromelanin, scientists are gaining a better understanding of the fundamental mechanisms behind PD. The development of new animal models that can produce human-like neuromelanin has been a game-changer, allowing researchers to observe how its accumulation leads to motor deficits, Lewy body-like inclusions, and neurodegeneration—all key features of PD [8]. This newfound knowledge opens entirely new avenues for possible treatment or even prevention procedures. Instead of just focusing on symptoms, researchers are now exploring strategies to modulate neuromelanin levels, such as slowing its age-related buildup, to prevent or delay the onset of neurodegeneration [9]. Moreover, the ability to study neuromelanin in a living human brain is a relatively recent development made possible by the newly available neuromelanin-sensitive magnetic resonance imaging (NM-MRI) technique [10]. This specialized MRI technique allows researchers to visualize and measure the amount of neuromelanin in specific brain regions. This tool is revolutionizing the field by providing a non-invasive way to serve, for example, as a potential biomarker for early disease diagnosis, even in the prodromal (pre-symptomatic) stage. Also, using NM-MRI, studies have shown that neuromelanin naturally accumulates with age, but then plateaus and may even decline in older age. Understanding this normal trajectory is critical for accurately diagnosing pathological changes. While the link to PD is the most prominent, neuromelanin research is also extending to other areas of brain health. Indeed, neuromelanin-sensitive MRI is being explored as a tool to understand and measure dopamine function in people with conditions like schizophrenia and substance use disorders. In addition, the knowledge gained from neuromelanin studies could also shed light on the broader processes of brain aging and the factors that contribute to cognitive decline and neuronal vulnerability.

This article aims to provide an overview of melanin and neuromelanin, integrating their fundamental biochemistry with the latest insights from omics research in human neurological conditions. We describe the genetic aspects of melanogenesis, comparing human and fungal pathways, and explore the convergent evolutionary strategies that utilize melanin for survival against harsh environmental insults, including ionizing radiation. Such comparative insights are actually relevant for a better understanding of the full spectrum of melanin’s biological importance, from fundamental cellular processes to implications and applications for human health in extreme environments, such as space.

## 2. Human Melanin and Neuromelanin

### 2.1. Melanogenesis

**Figure 1:**
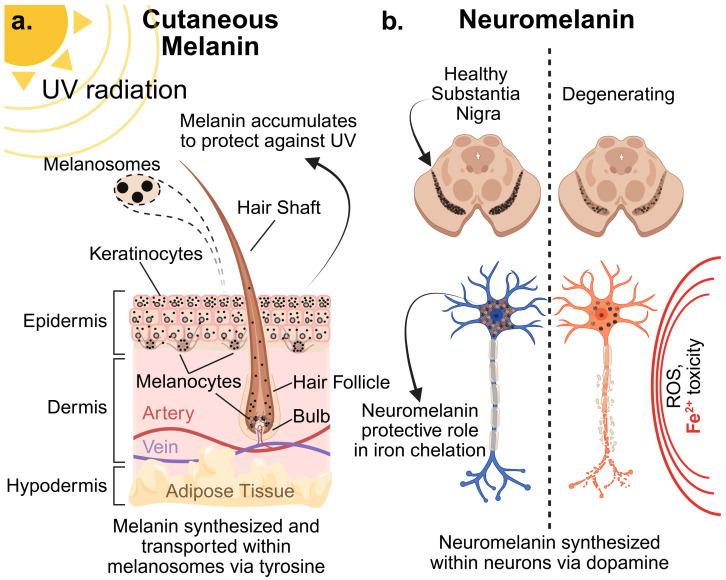
(**a**) Cutaneous melanin synthesis occurs in the skin, with epidermal melanocytes utilizing tyrosine pathways to deliver melanin-filled melanosomes to keratinocytes, with increasing levels of melanin deposited at more apical layers to maximize protection from UV radiation. This transferred melanin forms a protective shield around the keratinocytes’ nuclei to prevent DNA damage. Similarly, bulb melanocytes release melanosomes within the hair follicle, determining hair coloration [11]. (**b**) Conversely, melanocytes do not exist in the brain, with neuromelanin synthesized instead within neurons (typically catecholamine neurons of the substantia nigra or locus coeruleus) through dopaminergic pathways. This neuromelanin is thought to play an important role in iron chelation and protection from reactive oxygen species (ROS) damage. Dysregulation of neuromelanin function and associated neurodegeneration is considered crucial to the development of progressive disorders, including Parkinson’s Disease (PD). Created in BioRender. Hatch, K. (2025) https://BioRender.com/lx9p6qb (accessed on 15 November 2025).

Human melanin synthesis is a highly regulated process involving multiple genes, enzymes, and transport proteins. While the basic enzymatic machinery is well-characterized, the precise pathways and regulatory mechanisms distinguishing cutaneous melanin from neuromelanin are still subjects of intense research (Figure 1).

Skin and hair pigmentation in humans is primarily attributed to two types of melanin: eumelanin (black/brown) and pheomelanin (red/yellow). Their biosynthesis occurs within melanocytes and is largely controlled by a cascade of enzymatic reactions. The central enzyme in this pathway is tyrosinase (*TYR*), a copper-containing monooxygenase. However, the genetic blueprint for cutaneous melanogenesis involves several key genes:

- Tyrosinase (*TYR*): encodes the rate-limiting enzyme in melanin synthesis. Specifically, *TYR* catalyzes the hydroxylation of L-tyrosine to L-3,4-dihydroxyphenylalanine (L-DOPA) and the oxidation of L-DOPA to DOPAquinone. Genetic mutations in TYR are the primary cause of oculocutaneous albinism type 1 (*OCA1*) [12].
- Tyrosinase-Related Protein 1 (*TYRP1*)/DOPAchrome Tautomerase (*DCT*): encoded by *TYRP1* and *DCT*, respectively, these enzymes catalyze subsequent steps in the eumelanin pathway. *TYRP1* oxidizes 5,6-dihydroxyindole-2-carboxylic acid (DHICA) and stabilizes tyrosinase. *DCT* catalyzes the tautomerization of DOPAchrome to DHICA. Mutations in *TYRP1* cause oculocutaneous albinism type 3 (*OCA3*), and DCT mutations are linked to certain pigmentation disorders [13].
- Melanocortin 1 Receptor (*MC1R*): This G protein-coupled receptor, expressed on melanocytes, plays a pivotal role in regulating the type of melanin produced. Upon binding of its ligand, alpha-melanocyte-stimulating hormone (α-MSH), *MC1R* activates adenylate cyclase, leading to increased intracellular cAMP levels. High cAMP levels promote eumelanin synthesis, while low cAMP levels favor pheomelanin synthesis. Common genetic variants in *MC1R* are strongly associated with red hair, fair skin, and increased risk of melanoma [14].
- Oculocutaneous Albinism Type 2 (*OCA2*): Encodes the P protein, a melanosomal transmembrane protein thought to be involved in tyrosine transport into melanosomes or regulation of melanosomal pH. Mutations in *OCA2* are the most common cause of OCA2 [15].
- *SLC45A2* (Membrane-Associated Transporter Protein, MATP): This gene encodes a transporter protein likely involved in melanosome biogenesis or transport of melanin precursors. Mutations in *SLC45A2* cause oculocutaneous albinism type 4 (OCA4) [16].
- Microphthalmia-associated Transcription Factor (*MITF*): Considered the “master regulator” of melanogenesis, *MITF* controls the expression of *TYR, TYRP1, DCT*, and other genes involved in melanocyte development, survival, and melanin synthesis. Genetic variations in *MITF* can lead to diverse pigmentation and developmental defects [17]. In general, the genetic regulation of cutaneous melanin is tightly controlled by signaling pathways (e.g., Wnt/β-catenin, MAPK/ERK, cAMP/PKA) that converge on *MITF*, ensuring coordinated production and deposition of melanin within melanosomes.

### 2.2. Melanin in Human Health and Disease

The primary purpose of melanin in humans is to provide pigmentation to skin, hair, and eyes for protection against ultraviolet (UV) radiation from sun exposure [18]. Melanin is produced by melanocytes, which form moles when clustered, in contrast to ephelides, or freckles, which are patches of higher melanin concentrations that darken with increased sun exposure. Melanin loss or degeneration has even been implicated as a contributing risk factor in several ocular and auditory disorders, where its presence in the iris and cochlear tissue is thought to provide a protective role [19]. As previously mentioned, there are two main types of melanin. Eumelanin, which is primarily composed of 5,6-dihydroxyindole (DHI) and DHICA units, is more effective in the absorption and neutralization of harmful UV rays than pheomelanin (which consists of sulfur-containing benzothiazine and benzothiazole derivatives), thus eumelanin provides greater photoprotection [20]. Indeed, eumelanin possesses antioxidant and free radical scavenging properties, while pheomelanin is thought to promote the formation of free radicals and may, in fact, be phototoxic [21]. Research has shown several instances where these specific properties (or loss of their function) can contribute to the progression of melanin-associated diseases.

#### 2.2.1. Skin Cancer

Skin cancer has a higher prevalence among lighter skinned individuals who not only have less overall melanin than those with darker skin, but also a lower proportion of eumelanin to pheomelanin [22]; higher proportions of pheomelanin are prevalent among people living at higher latitudes with fair hair and skin tones, likely for the evolutionary purpose of permitting increased skin penetration of UV rays to facilitate sunlight-dependent biological processes, like Vitamin D production, in geographical regions with lower levels of sun exposure [21]. Pheomelanin synthesis depletes reactive oxygen species (ROS) scavengers and makes melanocytes more susceptible to DNA damage, which may contribute to the development of skin cancer.

#### 2.2.2. Albinism

The congenital absence (or reduction) of melanin is known as albinism. There are two primary forms: oculocutaneous albinism affects the skin, hair, and eyes, while ocular albinism is less common and affects the eyes with minimal changes to hair or skin. These disorders are the result of genetic mutation and often lead to eye problems like light sensitivity, nystagmus, strabismus, and blurred vision [23]. There is some evidence that albinism is associated with neurodevelopmental delays or impairment [24], but it is unclear how the genetic mutations causing impaired cutaneous melanin production could potentially impact neuromelanin expression in the brain. Some rare disorders, like Chediak-Higashi syndrome, involve the presentation of partial albinism along with neurodegenerative symptoms [25] (including Parkinsonian presentations in severe cases [26]), but any relationship between these manifestations is unclear.

#### 2.2.3. Vitiligo

Loss of pigment due to the autoimmune disorder vitiligo occurs when melanocytes die or stop producing melanin, resulting in patches of depigmented skin. This maladaptive autoimmune response is orchestrated by local autoreactive cytotoxic T-cell engagement with melanocytes, activity that is mediated by interferon gamma signaling and involves a positive feedback loop with surrounding keratinocytes and locally secreted chemokines [27]. It is interesting to note that vitiligo is associated with decreased risk of developing Parkinson’s Disease, but for those with vitiligo who do develop PD, there is an elevated mortality rate and greater prevalence of cardiometabolic comorbidity [28]. Further research is needed to determine if perhaps the autoimmune activity against melanin in vitiligo may extend to neuromelanin in PD, promoting accelerated disease progression.

### 2.3. Neuromelanin Biosynthesis: A Distinct, Age-Dependent Pathway

**Figure 2:**
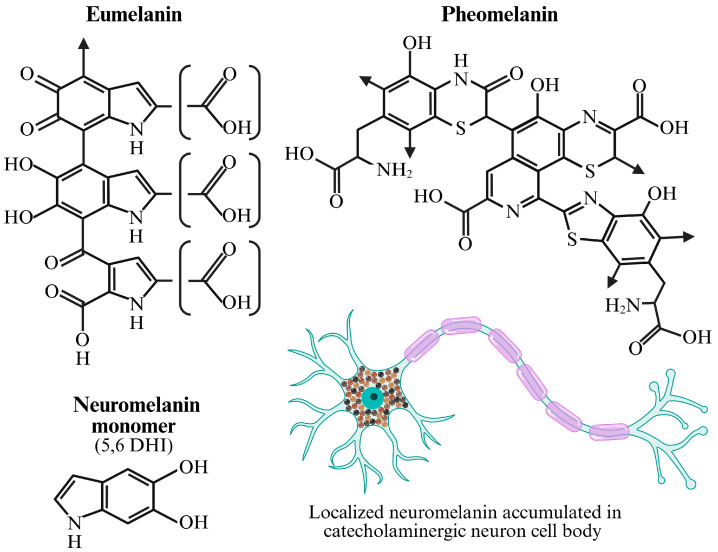
This figure illustrates the chemical diversity among the major melanin pigments found in humans and highlights the specific cellular context of neuromelanin. Eumelanin (**Top Left**): The structure shown represents a proposed oligomer of eumelanin, the dark brown-to-black pigment responsible for dark hair, skin, and eye color. It is a heterogeneous polymer composed primarily of cross-linked 5,6-dihydroxyindole (DHI) or 5,6-dihydroxyindole-2-carboxylic acid (DHICA) units. This highly conjugated and insoluble structure is exceptionally effective at absorbing a broad spectrum of ultraviolet (UV) radiation, providing significant photoprotection. Pheomelanin (**Top Right**): This structure represents a proposed model for pheomelanin, the lighter red-to-yellow pigment prevalent in red hair and fair skin. Its distinct coloration and chemical properties are due to the incorporation of sulfur, which originates from the amino acid cysteine reacting with dopaquinone during synthesis. The resulting benzothiazine and benzothiazole units create a more irregular, less-polymerized structure that is less efficient at UV absorption and can even produce ROS upon UV exposure. Neuromelanin Monomer (**Bottom Left**): The molecule 5,6-dihydroxyindole (5,6 DHI) is shown as a primary building block of neuromelanin. Unlike cutaneous melanins, which are synthesized enzymatically in melanosomes, neuromelanin is an auto-oxidative byproduct that accumulates over a lifetime. It is formed from the non-enzymatic oxidation and polymerization of catecholamines, primarily dopamine, in the cytoplasm of specific neurons. Neuromelanin Localization (**Bottom Right**): The diagram of the neuron illustrates the characteristic accumulation of neuromelanin granules within the cell body (soma) of catecholaminergic neurons [29], such as those in the substantia nigra region of the brain. This pigment is not packaged in melanosomes but is contained within autophagic vacuoles. It plays a dual role: it is considered neuroprotective by chelating potentially toxic redox-active metals like iron, but its presence is also a hallmark of the neurons that degenerate in Parkinson’s disease. Chemical structures adapted from [32]. Created in BioRender. Hatch, K. (2025) https://BioRender.com/scqqfqv (accessed on 15 November 2025).

Neuromelanin pigmented granules accumulate in catecholaminergic neuronal cell bodies, composed primarily of protein complexes of eumelanin and pheomelanin [29] (Figure 2). Unlike the highly enzymatic and tyrosinase-dependent process of cutaneous melanogenesis, neuromelanin synthesis is believed to occur primarily through the autoxidation of cytosolic catecholamines, primarily dopamine and norepinephrine, in the presence of ferrous iron and other metals [30]. While tyrosinase is expressed in melanocytes, it is largely absent or expressed at very low levels in the substantia nigra and neurons involved in neuromelanin synthesis. Among others, the key differences and genetic implications for neuromelanin biosynthesis are:

- Precursors. Basically, neuromelanin is derived from dopamine and norepinephrine, which are neurotransmitters synthesized by tyrosine hydroxylase (*TH*) and dopamine β-hydroxylase (*DBH*), respectively [31]. Genetic variations in *TH* and *DBH* can influence the availability of these precursors, though their direct impact on neuromelanin levels is not fully understood.

- Non-Enzymatic Oxidation. Indeed, the inherent susceptibility of catecholamines to autoxidation in the presence of oxygen and redox-active metals like iron (Fe^2+^) is central to neuromelanin formation [33]. This process generates ROS and various quinones that polymerize to form neuromelanin.
- Enzymatic Contribution. While not a classical tyrosinase-driven process, some enzymes may still contribute to neuromelanin synthesis. For instance, aldehyde dehydrogenases (ALDHs) detoxify reactive aldehydes generated during catecholamine metabolism. Polymorphisms in *ALDH2* have been linked to PD risk and may indirectly affect neuromelanin accumulation by altering the balance of catecholamine metabolites [34]. Furthermore, evidence suggests that lysosomal enzymes and even low levels of non-melanogenic peroxidases within neurons might play a role in shaping neuromelanin aggregates [35].
- Lysosomal Involvement. Neuromelanin granules are enclosed within specialized lysosome-like organelles, often referred to as neuromelanosomes or autolysosomes. Genes involved in lysosomal biogenesis, autophagy, and protein degradation pathways (e.g., *GBA, LRRK2, PINK1, PRKN*) are critical for neuronal health and are frequently implicated in PD. Dysfunction in these pathways could impair neuromelanin granule turnover or lead to the release of potentially toxic neuromelanin components [36].
- Iron Metabolism. Iron is intimately associated with neuromelanin, and genes regulating iron homeostasis (e.g., *FTL* for ferritin light chain, *SLC11A2* for DMT1, *HFE* for hemochromatosis gene) are crucial. Dysregulation of iron metabolism, influenced by genetic factors, can exacerbate oxidative stress and influence neuromelanin aggregation and toxicity [37].

Moreover, intriguingly, while skin melanogenesis is an important protective mechanism against UV radiation, neuromelanin’s function appears more complex, serving as a sink for harmful substances and potentially modulating neuronal excitability, but also acting as a source of oxidative stress when its capacity is overwhelmed or when neuromelanin-containing neurons degenerate. Importantly, then, the genetic factors influencing neuromelanin are therefore intertwined with genes governing catecholamine synthesis, iron handling, lysosomal function, and oxidative stress responses. (Box 1).

Box 1 **Testable hypotheses and predictions.**

**Hypothesis H1** (Iron valence window): neuromelanin is maximally protective within a bounded Fe(II)/Fe(III) ratio; excess Fe(III) promotes Fenton chemistry and toxicity.

**Hypothesis H2** (Lipid co-polymer effect): Specific lipid adducts (e.g., polyunsaturated vs. saturated) shift neuromelanin’s redox potential and radical lifetime.

**Hypothesis H3** (Catechol precursor imprinting): Dopamine- vs. norepinephrine-derived polymers exhibit distinct metal-binding affinities and MRI contrast signatures.

**Hypothesis H4** (Lysosomal throughput): Enhancing autophagic flux reduces neuromelanin-associated α-synuclein aggregation and microglial activation after neuronal death.

**Hypothesis H5** (Metal chelation timing): Chelators are beneficial only when applied before lysosomal failure; late chelation mobilizes redox-active iron and worsens injury.

** **

Five distinct hypotheses designed to probe the complex functions of neuromelanin, exploring its protective versus toxic roles through an “iron valence window” (*H1*); the modulatory effect of lipid adducts (*H2*); the impact of its catecholamine precursors (*H3*); its interplay with lysosomal autophagy (*H4*), and the critical timing of therapeutic metal chelation (*H5*).

### 2.4. In Vivo and Ex Vivo Neuromelanin Detection Methods

The methods to detect NM are broadly categorized into non-invasive in vivo techniques applicable to living subjects and definitive post-mortem analyses performed on brain tissue.

#### 2.4.1. In Vivo Detection Methods:

Historically, in vivo detection of neuromelanin has been challenging, with specialized magnetic resonance imaging (MRI) techniques developed in the early 2000s, and positron emission tomography (PET) tracers developed only about a decade ago. While highly valued for their non-invasive capacity to assess neuromelanin at a clinical level, these methods are still limited in their sensitivity.

- MRI. The current gold standard for non-invasive NM detection is neuromelanin-sensitive MRI (NM-MRI). This specialized imaging technique does not visualize the NM pigment directly but rather a key component associated with it: iron. Neuromelanin has a high capacity to chelate, or bind, metals, particularly ferric iron (Fe^3+^) [7]. This NM-iron complex is paramagnetic, meaning it alters the local magnetic field [38]. This property directly influences the relaxation times of nearby water protons, which is the basis of the MRI signal. Specifically, the paramagnetic NM-iron complex causes a significant shortening of the longitudinal relaxation time, known as the T1 time. MRI sequences that are heavily T1-weighted, such as a two-dimensional fast spin-echo sequence, can capitalize on this effect. In these scans, tissues with a short T1 relaxation time appear bright (hyperintense). Consequently, the SNc and LC, which are rich in NM, are clearly visible as high-intensity regions against the surrounding brain tissue. The clinical utility of this technique is powerful; in patients with Parkinson’s disease, the volume and signal intensity of these bright regions are progressively reduced, correlating strongly with the severity of motor symptoms and the degree of underlying neuron loss [39]. While NM-MRI is an invaluable tool, it is an indirect measure, and the signal can be influenced by changes in iron concentration independent of NM. Therefore, standardized protocols for image acquisition and analysis are critical for reliable and comparable results across studies.
- PET. A more direct and quantitative in vivo method on the horizon is PET. This molecular imaging technique relies on the administration of a radioactive tracer (a radioligand) designed to bind to a specific molecular target in the body. For NM, the goal is to develop a radiotracer that can cross the blood–brain barrier and bind with high specificity and affinity to the pigment itself. Such a tool would provide a direct measure of NM concentration, potentially offering greater sensitivity than the indirect approach of MRI. While a dedicated NM-PET tracer is not yet in clinical use, several compounds have shown promise. For instance, some radioligands initially developed for imaging tau pathology, such as derivatives of phenyl/pyridinyl-isoquinoline, have demonstrated incidental binding to NM in human brain tissue [40]. This has spurred dedicated efforts to synthesize and validate novel tracers specifically for neuromelanin. A successful NM-PET tracer would represent a significant leap forward, enabling more precise quantification of neuronal loss and a more sensitive method for tracking disease progression and the effectiveness of neuroprotective therapies.

#### 2.4.2. Post-Mortem Detection Methods:

Post-mortem analysis of brain tissue provides the “gold standard” for confirming the presence of neuromelanin and validating findings from in vivo imaging. These methods offer unparalleled resolution and specificity.

- Histological Staining. The most fundamental post-mortem technique is direct visualization through histological staining. The Fontana-Masson stain is the classic and most specific method for this purpose [41]. This is an argentaffin reaction, where the reducing components within the melanin polymer directly reduce silver nitrate from the staining solution to black, metallic silver. This process stains NM granules a distinct dark brown or black, making them easily identifiable within the cytoplasm of neurons under a light microscope [42]. While other general stains like hematoxylin and eosin (H&E) can also reveal NM as coarse brown granules, the Fontana-Masson method provides superior specificity.
- Immunohistochemistry (IHC) and Immunofluorescence (IF). To understand which specific types of neurons contain NM and are degenerating, researchers use immunohistochemistry (IHC) or immunofluorescence. These techniques employ antibodies to label specific proteins. For example, an antibody against Tyrosine Hydroxylase (TH), the rate-limiting enzyme in dopamine synthesis, can be used to definitively identify dopaminergic neurons. By combining TH staining with the natural visibility of NM (or Masson-Fontana staining), one can confirm that the pigmented cells in the SNc are indeed dopaminergic and can quantify the loss of these specific cells in PD brains.
- Electron Microscopy (EM). For the highest possible resolution, electron microscopy (EM) is used to examine the ultrastructure of the NM granules themselves. EM reveals that NM granules are complex organelles, often containing a lipid core surrounded by a matrix of melanin, proteins, and lipids [43]. This high-magnification view allows researchers to study the morphology and composition of the granules, which may change in disease states, providing critical insights into the underlying cellular pathology of neurodegeneration.

In summary, the detection of neuromelanin is a multifaceted endeavor, with in vivo MRI providing a crucial window for clinical applications, while post-mortem techniques offer the definitive ground truth necessary for validating non-invasive methods and conducting fundamental research.

### 2.5. Neuromelanin in Human Health and Disease

The application of omics technologies has provided unprecedented molecular detail into the environment of neuromelanin-containing neurons, elucidating their vulnerabilities and unique molecular signatures in both healthy aging and various neurological conditions. Neuromelanin naturally accumulates with age, peaking in middle to late adulthood. This accumulation is associated with increased iron deposition in the substantia nigra and locus coeruleus. Omics studies in the aging brain reveal several key changes within neuromelanin-rich regions. We can now recognize different -omics aspects as follows:

- Transcriptomics. Aged substantia nigra neurons show increased expression of genes related to oxidative stress (e.g., *NQO1*, *HMOX1*), inflammation (e.g., complement components, microglial activation markers), and DNA damage response (*GADD45A/B*) [44]. There is also evidence of reduced expression of genes encoding mitochondrial function and protein quality control pathways, suggesting a decline in cellular resilience [45].
- Proteomics. Proteomic analyses of aged substantia nigra tissue and isolated neuromelanin granules reveal an accumulation of oxidatively damaged proteins, advanced glycation end products (AGEs), and lipofuscin-like aggregates. Metal-binding proteins, particularly ferritin, are also increasingly abundant, consistent with the age-dependent increase in iron within these regions [7].
- Epigenomics. Age-related epigenetic drift, characterized by global hypomethylation and site-specific hypermethylation, is observed in neuromelanin-containing neurons. These changes can alter the expression of genes involved in neuronal function, stress response, and inflammation, potentially priming these neurons for age-related decline and disease susceptibility [46]. For instance, differential methylation of genes involved in dopamine synthesis or synaptic function may contribute to subtle cognitive shifts seen in healthy aging.

#### 2.5.1. Parkinson’s Disease (PD)

PD is characterized by the progressive degeneration of pigmented dopaminergic neurons in the substantia nigra pars compacta, leading to canonical motor symptoms, as well as the pigmented noradrenergic neurons of the locus coeruleus and dorsal motor nucleus of the vagus, leading to typical non-motor symptoms [4,47]. The macroscopic depigmentation of the substantia nigra is a classical pathological feature, reflecting the loss of neuromelanin-containing neurons. Omics studies have significantly advanced our understanding of neuromelanin’s role in PD. We can recognize the following findings across each specific type of -omic approach, namely:

- Transcriptomics. Single-cell RNA sequencing (scRNA-seq) has revolutionized our ability to study specific neuronal populations. In PD, scRNA-seq of substantia nigra neurons reveals a selective downregulation of genes critical for dopaminergic identity and function (e.g., *TH*, *SLC6A3* for dopamine transporter, *ALDH1A1* for aldehyde dehydrogenase) in vulnerable neuromelanin-rich neurons [48]. Concurrently, there is an upregulation of genes associated with inflammation (*HLA-DRB1*, *CD68*), ER stress, and unfolded protein response. Genes related to iron metabolism, such as FTL (ferritin light chain) and *SLC11A2* (*DMT1*), show altered expression, reflecting iron dyshomeostasis in the degenerating substantia nigra [49].
- Proteomics. Proteomic analyses of post-mortem substantia nigra tissue from PD patients consistently show an enrichment of oxidatively modified proteins, lipid peroxidation products, and aggregated α-synuclein. Neuromelanin has been shown to physically interact with and potentially sequester α-synuclein, a key protein in Lewy body pathology [50]. While this might initially be protective, the interaction can also lead to the formation of more toxic, aggregated species when neuromelanin’s buffering capacity is overwhelmed. Lysosomal proteins, essential for neuromelanin granule turnover, also show altered levels, consistent with lysosomal dysfunction being a core component of PD pathogenesis.
- Epigenomics. Epigenetic studies in PD patients reveal widespread DNA methylation changes in the substantia nigra, particularly in promoter regions of genes involved in mitochondrial function, lysosomal pathways, and inflammation. For example, hypermethylation of the *SNCA* promoter (encoding α-synuclein) can reduce its expression, yet abnormal methylation patterns elsewhere may contribute to disease progression [51]. Histone modification patterns (e.g., acetylation, methylation) are also altered, impacting chromatin accessibility and gene expression in neuromelanin-vulnerable neurons.
- Metabolomics. Metabolomic profiling of CSF and brain tissue in PD indicates dysregulation of catecholamine metabolism. Elevated levels of dopamine oxidation products and decreased levels of protective antioxidants suggest increased oxidative stress directly related to neuromelanin synthesis and breakdown. Changes in iron-related metabolites and lipid profiles also point towards lipid peroxidation, a process exacerbated by iron-laden neuromelanin [52].
- Genetic Intersections with PD Risk Genes. Curiously, many genes linked to familial PD, such as leucine-rich repeat kinase 2 (*LRRK2*), alpha-synuclein (*SNCA*), PTEN-induced putative kinase 1 (*PINK1*), parkin (*PRKN*), and glucocerebrosidase (*GBA*), play roles in lysosomal function, mitochondrial quality control, and α-synuclein handling. Mutations in these genes can indirectly impact neuromelanin accumulation and turnover, contributing to the vulnerability of neuromelanin-containing neurons. For example, *GBA* mutations, which cause Gaucher disease, are a significant risk factor for PD, and *GBA* enzyme deficiency affects lysosomal function and lipid metabolism, potentially exacerbating neuromelanin-related toxicity [36].

Integrating single-cell transcriptomics, proteomics of neuromelanin granules, metabolomics, and epigenomics supports the following model: that vulnerable neurons of the substantia nigra pars compacta with high catecholamine flux accumulate iron-laden neuromelanin within compromised lysosomal systems. When autophagic/lysosomal throughput or iron buffering is exceeded, oxidative chemistry around neuromelanin intensifies, promoting α-synuclein misfolding/aggregation and microglial activation upon neuromelanin release. This places neuromelanin at the center of a homeostatic axis: protective when buffered, pathogenic when the redox and degradative capacities are saturated.

#### 2.5.2. Alzheimer’s Disease (AD)

While PD primarily affects the substantia nigra, Alzheimer’s disease (AD) pathology impacts different brain regions, though the locus coeruleus, rich in noradrenergic neuromelanin-containing neurons, is an early and consistently affected area. Among the main -omics findings, we have recognized the following ones:

- Transcriptomics. In early AD, neurons show transcriptional downregulation of genes related to noradrenergic biosynthesis (e.g., *DBH*, *TH*) and an upregulation of genes associated with inflammation and stress responses [53]. This suggests that degeneration, beginning even before widespread amyloid/tau pathology, contributes to the cognitive and behavioral symptoms of AD.
- Proteomics. Proteomic studies in AD brains indicate that neuromelanin granules may co-localize with or bind to early aggregates of amyloid-beta (Aβ) and hyperphosphorylated tau [54]. While this binding might initially be a protective sequestration mechanism, it could also contribute to the local neuroinflammation and neuronal death if the neuromelanin-amyloid-β/tau complex becomes toxic.
- Epigenomics. Like PD, epigenomic changes in AD brains, particularly in the hippocampus and cortex, include altered DNA methylation patterns that could influence the expression of genes related to neuronal resilience, inflammation, and AD-specific protein pathology [55].

#### 2.5.3. Multiple System Atrophy (MSA)

MSA is a synucleinopathy characterized by glial cytoplasmic inclusions. While neuromelanin loss is less prominent than in PD, the substantia nigra neurons are still affected. Omics approaches highlight the critical role of glial-neuronal interactions. Transcriptomics studies in MSA brains show upregulation of oligodendroglial stress genes (*PLP1*, *TPPP*) and microglial activation markers near neuromelanin-containing regions, suggesting that neuromelanin-related oxidative stress may prime glia in the surrounding tissue, contributing to the overall neuroinflammatory milieu [56].

#### 2.5.4. Schizophrenia and Other Neuropsychiatric Disorders

Dysregulation of dopamine systems is a key feature of schizophrenia. While neuromelanin is less directly implicated, altered dopamine metabolism can indirectly impact neuromelanin synthesis and accumulation. Postmortem studies using visual assessment have reported reduced pigmentation in the substantia nigra of some schizophrenia patients, though this is not a consistent finding across all studies [57]. Metabolomics studies of CSF and brain tissue have shown altered levels of dopamine metabolites (e.g., homovanillic acid (HVA) and 3,4-dihydroxyphenylacetic acid (DOPAC)), which could reflect changes in neuromelanin turnover or dopamine buffering capacity in these conditions [58]. The exact link between neuromelanin and neuropsychiatric disorders remains an area for further omics-driven research.

#### 2.5.5. Amyotrophic Lateral Sclerosis (ALS)

While not classically associated with neuromelanin loss, recent studies suggest that iron accumulation and oxidative stress, which are intimately linked to neuromelanin, play a role in motor neuron degeneration in ALS [59]. While direct neuromelanin involvement is minimal, the shared pathways of metal dyshomeostasis and oxidative stress suggest potential, though indirect, connections that omics studies are beginning to explore.

#### 2.5.6. Neuroinflammation and Immune Responses

A critical emerging aspect of neuromelanin pathology is its role in neuroinflammation. Neuromelanin, when released from dying neurons or present in excess, can act as a “damage-associated molecular pattern” (DAMP), triggering innate immune responses. Newer omics analyses informed us about the following:

- Transcriptomics. Studies show that microglia exposed to synthetic or isolated neuromelanin express pro-inflammatory genes (e.g., *IL1β*, *TNF*, *NOS2*) and downregulate anti-inflammatory markers [60]. This shift in microglial phenotype contributes to chronic neuroinflammation in conditions like PD.
- Proteomics. The proteome of neuromelanin-stimulated microglia reveals an activation of pathways related to antigen presentation (MHC-I/II), phagocytosis, and inflammasome activation. Oxidized proteins and lipids associated with neuromelanin granules can further exacerbate the inflammatory cascade [61].
- Genetic Predisposition. Genetic variants in immune-related genes (e.g., *HLA* locus, *TREM2*, *LRRK2*) can modulate the brain’s inflammatory response to neuromelanin and other DAMPs, influencing disease susceptibility and progression [62].

## 3. A Special Case: Fungal Melanin as a Comparative Model System (Leveraging Fungal Melanogenesis to Probe Neuromelanin)

Melanin is widespread in fungi, where it provides crucial protection against a variety of environmental stressors, including UV radiation, desiccation, enzymatic degradation, and host immune defenses in pathogenic species. Unlike human neuromelanin, fungal melanin is typically found in the cell wall, acting as a robust protective barrier [63]. The biosynthesis of fungal melanin is genetically diverse, involving distinct pathways and enzymes.

Fungal melanins may provide experimentally tractable surrogates to interrogate principles that are challenging to resolve in human brain tissue. For example, the laccase-driven melanization of *Cryptococcus neoformans* polymerizes dopamine, L-DOPA, and norepinephrine, relevant precursors for neuromelanin—allowing systematic control of metal stoichiometry (Fe/Cu), lipid cofactors, and quinone intermediates. Raman/EPR/solid-state nuclear magnetic resonance (NMR) spectroscopy and electrochemical assays can then quantify how these variables tune radical density, charge transport, and metal valence cycling. Standardized dopamine-melanin preparations with defined Fe(II)/Fe(III) ratios and lipid admixtures can help map phase diagrams of protective versus pro-oxidant behavior. Such datasets can guide the interpretation of neuromelanin-MRI signals and inspire chelation or lysosomal modulation strategies in vivo.

### 3.1. Genetic Pathways of Fungal Melanogenesis

Fungi synthesize melanin primarily through two major pathways, often referred to as the DOPA-melanin pathway and the dihydroxynaphthalene (*DHN*)-melanin pathway. A third pathway, involving gamma-glutaminyl-4-hydroxybenzene (*GHB*) melanin, is also known [64].

#### 3.1.1. DOPA-Melanin Pathway (Tyrosinase/Laccase Dependent)

This pathway is common in pathogenic fungi like *Cryptococcus neoformans* and *Aspergillus niger*. It utilizes phenolic substrates like L-DOPA, tyrosine, or catecholamines, like human melanogenesis precursors, but involves different enzymes. The genes involved are, among others, *TYR* (as in *Aspergillus nidulans*) and *LAC1* (as in *C. neoformans*). These genes encode tyrosinases or laccases, which are copper-containing enzymes that oxidize phenolic precursors (e.g., L-DOPA) to quinones. In *C. neoformans*, the *LAC1* gene encoding laccase is essential for melanin synthesis and virulence [65].

Multicopper Oxidase Genes (e.g., MCO genes). These enzymes are crucial for oxidizing a wide range of substrates, including those involved in melanin synthesis. Specifically, DOPA is oxidized to DOPAquinone by tyrosinases or laccases. DOPAquinone then undergoes spontaneous cyclization and polymerization to form melanin. The expression of these genes is often regulated by environmental cues such as nutrient availability (e.g., nitrogen starvation) and temperature, often through transcription factors like *PKA* (protein kinase A) signaling pathway components in *C. neoformans* [66].

#### 3.1.2. Dihydroxynaphthalene (DHN)-Melanin Pathway (Polyketide Synthase Dependent)

This is the most common melanin pathway in filamentous fungi, including plant pathogens like *Magnaporthe oryzae* and human pathogens like *Aspergillus fumigatus*. This pathway does not use L-DOPA as a direct precursor. In particular, the genes involved are:

- Polyketide Synthases (PKS) (e.g., *PKS1* in *M. oryzae*, ALB1 in *A. fumigatus*). These are large, multi-domain enzymes that catalyze the initial steps of the pathway, producing 1,3,6,8-tetrahydroxynaphthalene (T4HN) from acetyl-CoA and malonyl-CoA.
- Reductases (e.g., 4HNR for 4-hydroxynaphthalene reductase). Catalyze reduction steps within the pathway.
- Dehydratases (e.g., SCD for scytalone dehydratase). Catalyze dehydration steps.
- Laccases (e.g., *LAC* in *A. fumigatus*). These enzymes act at the final step, polymerizing DHN precursors (e.g., 1,8-dihydroxynaphthalene, DHN) to form melanin.

**Figure 3:**
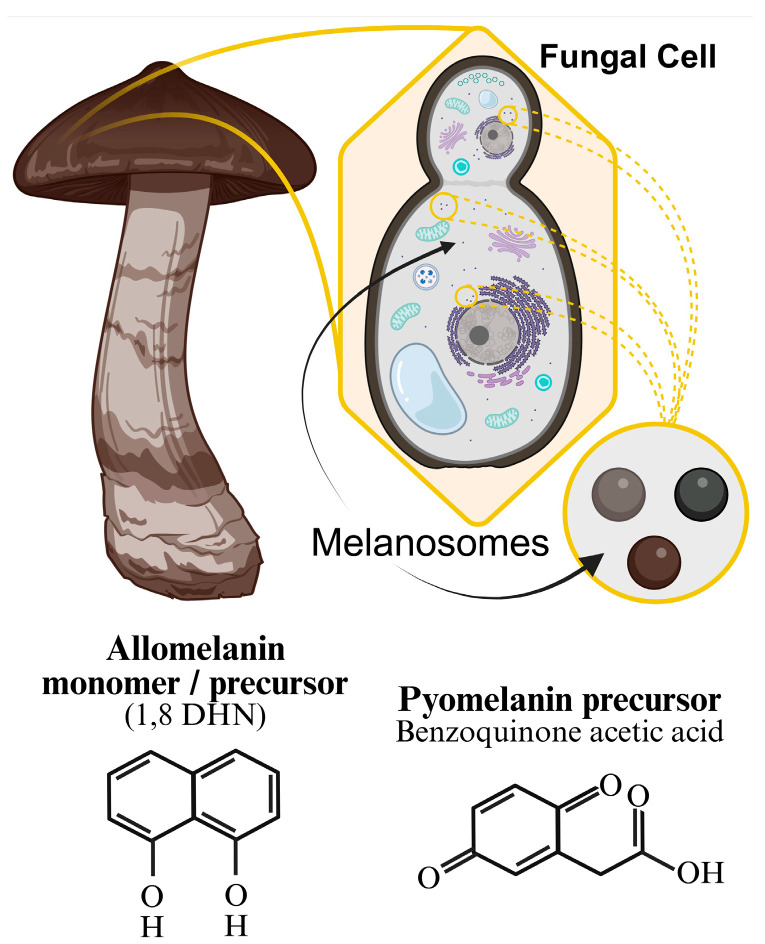
This figure illustrates some of the chemical diversity among melanin pigments found in fungi, highlighting the specific cellular context of melanin. Fungal melanin is produced within melanosomes inside fungal cells, transporting melanin to the cell wall and throughout the cytoplasm (**Top**). While eumelanin is produced by some fungi (Figure 2), DHN-melanin (allomelanin) (**Bottom Left**) [68] is by far the most common form of fungal melanin. Pyomelanin, the smallest of the melanin pigments, is particularly suited to covering cell surfaces. It undergoes a unique synthesis process that begins with tyrosine, as eumelanin does, but instead of following the homogentisate (HGA) pathway, it utilizes the precursor benzoquinone acetic acid (**Bottom Right**). Created in BioRender. Hatch, K. (2025) https://BioRender.com/bui29x3 (accessed on 15 November 2025).

Here, the pathway typically starts with a *PKS* producing naphthopyrone intermediates, leading to T4HN, which is then sequentially processed by reductases and dehydratases to form DHN. DHN is finally polymerized by laccases to yield DHN-melanin [63]. DHN-melanin pathway genes are often clustered in fungal genomes and are coordinately regulated by transcription factors (e.g., multi-drug resistance regulator 1 (*MRR1*) in *A. fumigatus*) in response to environmental stress or host signals [67]. (Figure 3).

#### 3.1.3. Gamma-Glutaminyl-4-Hydroxybenzene (GHB) Melanin Pathway

Found in certain fungi, such as *Agaricus bisporus*, this pathway uses gamma-glutaminyl-4-hydroxybenzene as a precursor, which is oxidized by tyrosinases [69]. This pathway is less common and less understood in the context of extremophile fungi compared to DOPA- and DHN-melanin.

### 3.2. Functional Roles and Extremophile Adaptation

Fungal melanin plays crucial roles in virulence and environmental persistence, making melanized fungi highly resilient. The genetic basis of these functions is directly linked to the robust and regulated synthesis of melanin.

#### 3.2.1. Virulence Factor

In many pathogenic fungi (e.g., *C. neoformans*, *A. fumigatus*), melanin is a critical virulence factor. It protects fungal cells from host defenses, including phagocytosis by macrophages, killing by ROS and reactive nitrogen species (RNS) from immune cells, and antifungal drugs. Genetic disruption of melanin synthesis genes (e.g., *LAC1* in *C. neoformans*, *PKS1* in *A. fumigatus*) significantly attenuates virulence in animal models [70].

Of particular interest is how fungi can utilize these mechanisms of virulence to infect humans. Fungal infections of the central nervous system can be severe, though often opportunistic, causing a range of ailments from stroke and meningitis to inflammatory exacerbation of non-infectious neurodegenerative diseases, including PD and AD [71]. For instance, if *C. neoformans* is not contained by the host immune system to the lungs, it can spread across the blood–brain barrier, and while fungal melanin in the cell wall may attract macrophage and microglia activity, if the fungus survives (sometimes even within the immune cells) it can subsequently lead to further melanin/neuromelanin production in the brain which is thought to possibly contribute to neurodegenerative pathogenesis [72,73]. Similarly, *C. albicans* can break down the blood–brain barrier, invading the brain and causing AD-like pathology through the generation of Aβ-like peptides; the efficient clearance of *C. albicans* from the brain by microglia activation mechanisms is crucial to prevent/reverse induced memory deficits [74,75].

#### 3.2.2. Stress Resistance

Melanin provides broad-spectrum protection against various environmental stresses:

- Mechanical. Melanin is thought to contribute to fungal cell wall rigidity, promoting survival in extreme low- and high-pressure environments, such as those conditions found in space and deep-sea hydrothermal vents [76,77]. There is also some evidence that melanin protects fungi from lysis [78].
- UV Radiation. Melanin absorbs UV radiation, dissipating the energy as heat, thus protecting cellular components from damage. Genetic pathways for melanin synthesis are often up-regulated in response to UV exposure [79].
- Desiccation. The hydrophobic nature of melanin-containing cell walls helps prevent water loss, enabling survival in arid environments [80].
- Heavy Metals: Like neuromelanin, fungal melanin can chelate heavy metals, detoxifying the environment [81].
- Oxidative Stress. Melanin acts as an efficient free radical scavenger, neutralizing ROS generated by environmental insults or metabolic processes [18,82].

#### 3.2.3. Radioprotection and Radiotropism

**Figure 4:**
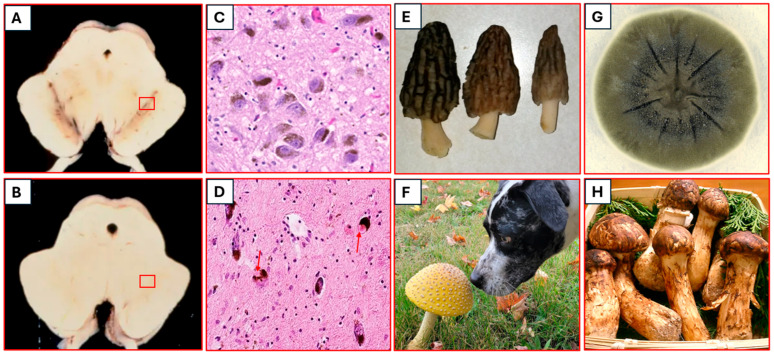
Melanin-Pigmented Cells Across Kingdoms: Same Functions and Radio-Absorbing Properties? (**A**–**D**) The presence (or absence) of neuromelanin-pigmented neurons (mostly dopaminergic neurons) anatomically aggregated in the region of the human brain called Substantia Nigra (nigra means black in Latin). (**A**) location (brainstem) and normal level of pigmentation of the SN; (**B**) almost total absence of neuromelanin-pigmented neurons in the brain of a subject with a clinical diagnosis of Parkinson’s disease (PD). (**C**,**D**) Comparison of neuromelanin-pigmented neurons from the corresponding normal (**A**) vs. PD brain (**B**). Red arrows in (**D**) indicate the presence of Lewy Bodies—a typical brain lesion associated with a clinical diagnosis of idiopathic PD—as detected by using hematoxylin and eosin stain (Lewy bodies are eosinophilic intracytoplasmic lesions). (**E**) *Morchella* mushrooms (commonly known as Morels), whose characteristic honeycomb caps owe their dark appearance to the presence of melanin-based pigment, in contrast to the mushroom example *Amanita muscaria* (**F**) which does not contain melanin but instead owes its complex pigmentation patterns to betaxanthins and a variety of amino acid derivatives (although the dog’s complex coat pigmentation patterns *are* the result of variations in cutaneous melanin distribution, similar to how human melanin is responsible for different skin tones and hair color). (**G**) a specific type of fungus, *Cladosporium sphaerospermum* (colony grown on potato dextrose agar, 14 days incubation), that has been found thriving in the high-radiation zone of the Chernobyl nuclear disaster site; its characteristic melanin-based black pigmentation is thought to contribute to this virulence through radiosynthesis mechanisms, converting the gamma radiation into biologically usable chemical energy. Interestingly, another fungus with remarkable radiation resilience, the *Tricholoma matsutake* mushroom (**H**), was anecdotally the first reported organism to grow in the devastated landscape of Hiroshima, Japan, after the 1945 atomic bombing; this fungus does not contain melanin, instead containing compounds that actually inhibit melanin production through disruption of tyrosinase activity. These mushrooms, rather than exhibiting the transformative radiotrophic properties of the melanin-rich *Cladosporium sphaerospermum* fungi, were particularly suited to the disturbed post-bombing landscape. Instead of converting radioactivity, these hardy fungi are uniquely adapted to survive the absorption and accumulation of radionucleotides from the soil, themselves becoming a source of radioactive contamination. External Image credit: Panel (**E**)—Debbie and Pete Guzek, cropped and used with permission; Panel (**G**)—“Cladosporium sphaerospermum colony” by Medmyco (original, uncropped work can be found at https://commons.wikimedia.org/wiki/File:Cladosporium_sphaerospermum_colony.jpg (accessed on 15 November 2025)) licensed under CC BY-SA 4.0 (To view a copy of this license, visit http://creativecommons.org/licenses/by-sa/4.0/ (accessed on 15 November 2025)); Panel—“Matsutake” by Tomomarusan (original, uncropped work can be found at https://commons.wikimedia.org/wiki/File:Matsutake.jpg (accessed on 15 November 2025)) licensed under CC BY 2.5 (to view a copy of this license, visit https://creativecommons.org/licenses/by/2.5/ (accessed on 15 November 2025)).

Perhaps one of the most astonishing functions of fungal melanin is its ability to protect against and even utilize ionizing radiation, including gamma radiation [83]. In terms of mechanisms, the following hypotheses and experimental findings have been described:

- Energy Dissipation. Melanin can absorb high-energy photons (gamma rays) and convert their energy into heat, minimizing direct damage to DNA and proteins [79].
- Electron Scavenging. Ionizing radiation produces free radicals and secondary electrons. Melanin’s unique electronic properties allow it to efficiently quench these highly reactive species [84].
- Redox Regulation. Melanin can serve as an electron donor/acceptor, buffering cellular redox states and protecting against oxidative damage induced by radiation [85].
- Radiotropism. Some melanized fungi, notably *Cladosporium sphaerospermum* isolated from the Chernobyl reactor, exhibit “radiotropism”—growth towards sources of ionizing radiation. This phenomenon suggests that melanin might not only protect but also mediate a form of radiation-induced metabolic activity, potentially utilizing the energy from radiation for growth or cellular processes [84]. The genetic basis for this radiotrophic growth is still under investigation, but it is likely to involve upregulation of melanin synthesis genes and stress response pathways, coupled with unique metabolic adaptations. Crucially, the radiotrophic capacity of highly melanized fungi like *Cladosporium sphaerospermum* at the Chernobyl exclusion zone and in space [86] to absorb ionizing radiation and convert it through radiosynthesis into chemical energy for biological processes (similar to how plants convert sunlight into energy through photosynthesis) is in stark contrast to other non-melanized fungi like *Tricholoma matsutake* (actually contains compounds that inhibit melanin production [87]) which have demonstrated a hardy resilience in highly radioactive environments (like post-war Hiroshima and Nagasaki and post-nuclear accident Fukushima in Japan [88]) but which absorb radiation (via radionucleotides from the soil) without converting it, becoming sources of radioactive contamination. This emphasizes the unique role of melanin in these complex adaptive and radioprotective mechanisms, which could be harnessed for human applications (Figure 4). Indeed, frogs at Chernobyl have demonstrated increased melanin production as an adaptive response to radiation, and human populations located in regions with high background radiation have demonstrated enhanced DNA repair capabilities, suggesting that harnessing biological adaptation (and evolutionary survival mechanisms) is possible and necessary for optimizing physiological response to extreme radiation environments [89].

The remarkable radioprotective capabilities of fungal melanin offer profound implications for human neuroprotection in high-radiation environments, such as space travel. Research into melanin-derived biomaterials for radiation shielding is a promising area, inspired by these extremophilic fungi [76].

## 4. Melanin Across Kingdoms

While humans and fungi are vastly different organisms, they share the trait of producing melanin, albeit through different genetic and biochemical pathways. This is a classic example of convergent evolution, where similar functional solutions arise from different evolutionary origins. Table 1 shows the breakdown of the functionally analogous genes and pathways for melanin synthesis across humans and fungi, while Table 2 shows a detailed comparison of melanin synthesis, regulation, and function. The pathways most significantly diverge at the point of melanin synthesis, using different enzyme families to achieve the polymerization of precursors into melanin. The expression and location of melanin synthesis are tightly controlled by specific regulatory genes. Despite distinct biosynthetic pathways and cellular localizations, neuromelanin in humans and melanin in fungi exhibit striking functional convergence. In both kingdoms, melanin is deeply connected to surviving environmental stress. Melanin and melanin-like pigments are found in a diverse array of cell types across multiple kingdoms, where they serve functions ranging from pigmentation and UV protection to defense and structural support (Table 3). This highlights a powerful evolutionary strategy: the use of complex, stable biopolymers for universal defense against diverse environmental and endogenous stressors.

Various properties of fungal melanin and human neuromelanin seem to be shared, such as:

- Polymeric Structure. Both are heterogeneous, amorphous polymers, making their precise chemical structures difficult to fully characterize.
- Dark Pigmentation. Both are dark brown to black, indicative of their extensive conjugated pi-electron systems, which are crucial for light/energy absorption.
- Radical Scavenging. Both are potent free radical scavengers, neutralizing ROS and protecting cells from oxidative damage.
- Metal Binding. Both have a high affinity for binding metal ions, particularly redox-active transition metals like iron and copper. This can be protective (detoxification) or potentially detrimental (catalyzing Fenton reactions in excess).
- Stress Resistance. Both contribute to resistance against various forms of stress, including oxidative, chemical, and potentially radiation stress.
- By contrast, some of the properties seem to diverge. Among the divergent properties, there are:
- Biosynthesis. Human cutaneous melanin relies on tyrosinase; neuromelanin largely on non-enzymatic autoxidation of catecholamines; fungal melanin employs distinct PKS, laccase, or tyrosinase pathways. The genes encoding these enzymatic machineries are unique to each type.
- Localization. Cutaneous melanin in melanocytes/melanosomes, neuromelanin in neuronal autolysosomes, fungal melanin predominantly in melanosomes and the cell wall.
- Age-Dependency. Neuromelanin accumulates with age; cutaneous and fungal melanin are typically synthesized on demand or as part of a developmental program.
- Radiotropism. Explicit radiotrophic growth has only been observed in certain melanized fungi, not in human cells or neurons.

This convergent evolution suggests that the fundamental chemical properties of melanin, irrespective of its precise biosynthetic origin, confer highly advantageous protective attributes. Understanding the genetic and molecular bases of these properties in fungi can provide bio-inspired solutions for human challenges, especially in areas like neuroprotection against cosmic radiation during long-duration space missions.

It may also be prescient to briefly discuss some of the similarities between the potential role of melanin in fungal and neuronal communication, an area of research that has only recently begun to develop. In the nervous system, neuromelanin facilitates neuronal signaling by contributing to neuroprotection through the sequestering of toxins and protecting neurons from damage like oxidative stress [92], and by acting as a neurotransmitter reservoir [93]. Evidence suggests neuromelanin can also facilitate effective dopamine function and stability by acting as a reservoir for lipids needed for local glycosylation [29], and intriguingly, its interactions with dopamine at the synaptic terminal suggest a possible mechanism by which dopamine neurons may be capable of a neuromelanin-mediated chemical memory [93]. Recent research has also found that depleted neuromelanin in the locus coeruleus of PD patients is negatively correlated with rhythmic alpha-band activity (8–12 Hz), which does not necessarily implicate neuromelanin as having a direct role in modulating cortical rhythmic activity but certainly associates the depletion of neuromelanin-positive neurons with altered synchronization of neuronal network oscillations [94].

Fungi, like neurons, use electrical signals to communicate. The role of melanin in this electrical transmission (usually through interconnected hyphae) is largely unexplored, but there has been interest in the exchange of electrical impulses between fungi and plants in their mycorrhizal networks to process ecological information [95], and some studies have identified possible contributions of melanin to this system of electrical signaling. It has been postulated that oscillations of extracellular electrical potentials between fungi colonies exhibit spiking characteristics that can be parsed into a communication not unlike human language [96]. Melanin may support this electrical communication by influencing conductivity within the hyphal and mycorrhizal network due to its semiconducting properties and chelation/sequestering of metal ions to enhance conductance of the fungal cell wall, almost acting like insulation, preventing electrical leakage akin to myelin along the neuronal axon [97,98]. Melanin is also known to be capable of transducing electromagnetic radiation into other forms of chemical and electrical energy, harvesting it for other biological/physiological processes; this is partially due to melanin’s semiconducting properties, which facilitate radiation absorption and conversion [99]. This unique capacity to harness austere environmental conditions is certainly an area of future biological and technological interest. (See Figure 4).

## 5. Future Directions and Possible Translational Applications

The comprehensive integration of omics data holds immense promise for deciphering the precise mechanisms by which neuromelanin modulates neuronal health and disease. Among the advanced omics technologies, the following ones seem to be the ones that will likely offer the highest informative value:

- Spatial Transcriptomics and Proteomics. These technologies will allow for the study of gene and protein expression within specific subregions of the substantia nigra and locus coeruleus, even down to individual neuromelanin-containing neurons, providing unprecedented spatial resolution of molecular changes in aging and disease [100].
- Single-Nuclei Multi-omics. Combining genomics, transcriptomics, and epigenomics from single nuclei can reveal the epigenetic regulatory landscapes that govern gene expression in neuromelanin-rich neurons, identifying novel targets for therapeutic intervention.
- Metabolomics of Neuromelanin Granules. More refined techniques to analyze the metabolic profiles directly from isolated neuromelanin granules could uncover novel biomarkers of oxidative stress or neurodegeneration.
- Non-invasive Imaging of Neuromelanin. Neuromelanin-sensitive MRI sequences (e.g., neuromelanin-MRI) are rapidly evolving as non-invasive tools to visualize and quantify neuromelanin levels in the substantia nigra and in vivo. These techniques can serve as early diagnostic biomarkers for PD, track disease progression, and monitor therapeutic responses. Further refinement and correlation with omics data will enhance their diagnostic and prognostic value [38].

### 5.1. Harnessing Melanin’s Multifaceted Functionality, Including Therapeutic Strategies Targeting Neuromelanin Pathways

Due to melanin’s unique biological properties and potential applications in medical and technological fields, considerable research is being performed to isolate and synthesize fungal melanin (particularly eumelanin) for repurposing [19,101,102]. In addition to the newer knowledge acquisition about melanin and neuromelanin, there is now the possibility to employ these new findings in possible therapeutic approaches, among them:

- Iron Chelation. Given the strong association between neuromelanin and iron, iron chelators are being explored as neuroprotective agents in PD to reduce oxidative stress [103].
- Modulation of Autophagy and Lysosomal Function. Strategies aimed at enhancing lysosomal function and autophagy could promote the clearance of damaged neuromelanin granules and α-synuclein aggregates, offering novel therapeutic avenues for PD.
- Melanin-Inspired Biomaterials. The remarkable radioprotective and transducing properties of fungal melanin are inspiring the development of novel biomaterials for radiation shielding [104] and sustainable bioelectronic applications [105]. This could range from melanin-coated surfaces in spacecraft to potential pharmaceutical interventions that enhance endogenous melanin’s protective capacities in astronauts or patients undergoing radiation therapy.
- Targeting Neuroinflammation. Given neuromelanin’s role as a DAMP, therapies aimed at modulating microglial activation and reducing neuroinflammation (e.g., through specific cytokine inhibitors or microglial repolarizing agents) could be beneficial in PD and other neurodegenerative conditions.
- Genetic Risk Factor Integration. Integrating genetic risk factors for PD and AD with neuromelanin-related omics data will help to identify individuals at higher risk for neurodegeneration and allow for personalized preventive or early intervention strategies. Understanding how common genetic variants (e.g., in GBA or LRRK2) impact neuromelanin biology is crucial.
- Comparative Biology for Human Benefit. Continued research into fungal melanin’s radioprotective mechanisms at a genetic and molecular level can provide blueprints for engineering human cells or developing targeted therapies to enhance neuroprotection against cosmic radiation during long-duration space missions, an increasingly critical challenge for space exploration. For instance, Antarctic black fungi (including the highly melanized *Cryomyces antarcticus*) have demonstrated resistance to simulated Martian conditions, exhibiting biological properties which could prove invaluable to future space exploration [106].

### 5.2. Fungal Melanin (And Maybe Someday, Neuromelanin) as a Bioavailable Radiation Countermeasure?

Fungal melanin is known to exhibit radioprotective and antioxidant, radical scavenging properties [107]; this has prompted some research into how natural or synthetic melanin may be utilized for the development of prophylactic and reparative treatment measures in response to radiation-induced gastrointestinal damage and even irritable bowel syndrome [108,109]. Microalgae-based oral microcarriers have been shown to promote gut microbiota homeostasis and intestinal protection in a preclinical model of cancer radiotherapy [110]; while the microalgae used (*Spirulina platensis*) does not itself produce melanin (although many microalgae do so, particularly species of cyanobacteria), it is rich in the precursor L-tyrosine which may inadvertently influence melanin production in the gut and elsewhere, possibly compounding the beneficial impact of the cryoprotective drug used in the study to reduce free radical damage from radiation therapy. The administration of allomelanin, a nitrogen-free melanin commonly found in fungi, has been shown to improve survival from and mitigate the effects of acute radiation syndrome when administered as a countermeasure shortly after exposure [111]. Indeed, melanin is known to survive the gut, and research has determined that ingestion of melanin-containing black mushrooms (or white mushrooms supplemented with melanin) can provide significant radioprotection in mice, acting as an internal shield to protect gastrointestinal tissue from the devastating effects of a lethal total body ionizing radiation dose [112]. The potential for repurposing melanin-rich mushrooms for orally administered radiation countermeasures is remarkable; when one considers the increasing prevalence of dietary supplements promoting the health benefits of mushrooms [113] (such as mushroom coffee, which attributes fruiting body mushroom extracts (which are rich in melanin and melanin precursors) for a purported, though unverified, “brainpower boost”), the capacity to widely disseminate such a countermeasure is truly exciting and could save countless lives. The further investigation into the possible effects of orally bioavailable melanin supplements to potentially influence the progression of neurodegenerative disorders seems a distant, though perhaps feasible, dream. Melanin nanoparticles are gaining popularity as a promising therapeutic tool in a variety of biomedical applications [114]. In addition to several utilizations in the field of radiation countermeasures, there has been some interest in the therapeutic potential of melanin nanoparticles administered to the brain for the treatment of ischemic stroke due to melanin’s natural scavenging activity for ROS and RNS [115]. It is possible that similar treatments may be developed in the future for neurodegenerative disorders, particularly those affecting neuromelanin-positive neurons. Even further, perhaps these therapeutic models could eventually lead to the administration of neuromelanin directly to the brain in a radioprotective capacity for long-duration space flight missions, where the effect of radiation on neuronal function is thought to be a highly prohibitive factor.

### 5.3. Metabolomic and Microbiomic Perspectives

Interestingly, there has been recent interest in the possibility that neuromelanin may act as a fuel source for cellular functioning, absorbing energy from electromagnetic radiation and supplementing this with ATP as a complementary supply [116]. This theory postulates that many neurodegenerative disorders may be influenced by metabolic failures directly relating to melanin/neuromelanin dysfunction. Perturbation of the gut microbiome and mycobiome has been linked to the development or resistance to metabolic diseases, and it has been noted that long-term dietary exposure to melanin-producing *Candida albicans* positively modulates metabolic hormones and the immune landscape [117,118,119]. The skin microbiome is known to influence cutaneous melanogenesis through inflammatory pathways and modulation of prebiotic and probiotic milieus; furthermore, increased inflammatory cytokine secretion (specifically interleukin 1β) via ultraviolet light exposure to the skin can promote melanogenesis in melanocytes [120,121]. Thus, it stands to reason that the gut microbiome and related inflammatory activity there may also influence melanogenesis throughout the body and brain, to the extent that these inflammatory signals and microbiomics changes can communicate with downstream pathways via the gut–brain (or gut–organ) axis. Gut dysbiosis and inflammation have been linked to neurodegenerative diseases like PD, which can involve the loss and dysfunction of neuromelanin-containing neurons. Typical treatment plans involve maintaining a healthy diet, and research has investigated how dietary changes (particularly increasing intake of antioxidant-rich foods) could influence PD progression [122,123]. In addition to inflammatory and immune-modulatory activity, emerging evidence suggests the gut microbiota plays a key role in maintaining adequate dopamine bioavailability in the brain [124], which could have huge implications for neuromelanin production and the subsequent progression (or regression) of neurodegeneration. Research has shown that mice and human survivors of radiation exposure exhibit distinct gut microbiota with enhanced tryptophan metabolic pathways, a non-canonical precursor of melanin [125,126]. Indeed, a large animal model of low-dose lower hemibody radiation found evidence of gut–brain axis involvement in dopaminergic signaling in proteomic profiles of the frontal cortex, which could perhaps influence melanogenesis pathways affecting other brain regions [127]. This field merits closer examination as we strive to understand the role of neuromelanin in the progression of neurodegeneration and how environmental factors may contribute.

## 6. Conclusions and Future Perspectives

Neuromelanin stands as a fascinating testament to evolutionary adaptation, playing a crucial yet complex role in human brain homeostasis. While its synthesis differs genetically and mechanistically from classical cutaneous melanogenesis, its deep involvement in metal chelation, redox buffering, and inflammatory modulation positions it at the nexus of neuroprotection and neurodegeneration. Omics technologies have been instrumental in unraveling the intricate molecular milieu of neuromelanin-containing neurons, revealing distinct transcriptional, proteomic, and epigenomic vulnerabilities that underpin their selective degeneration in conditions like PD and AD. Moreover, the comparative study of fungal melanin, with its genetically diverse biosynthetic pathways and remarkable capacity for radioprotection, offers compelling insights into the convergent evolution of protective biopolymers and provides bio-inspiration for novel therapeutic and protective strategies. In truth, the radioprotective properties of neuromelanin are as yet largely unknown. Indeed, this is one of several primary avenues of future research which we hope to encourage. Given the known and hypothesized parallels between the function of neuromelanin and fungal melanin, this presents a unique opportunity to utilize fungal melanin as a model system to increase our understanding of neuromelanin and thus identify potential areas where neuromelanin can be indeed used or improved for the advancement of human health, whether through enhanced treatments for disease or the development of cutting edge technology or techniques for future environmental exposure challenges (such as those faced during space exploration). As we continue to push the boundaries of space exploration and face increasing environmental stressors, a deeper understanding of melanin (and neuromelanin), from its fundamental genetic blueprints to its complex roles in human brain health and extremophile survival, remains paramount for advancing neurobiology and developing innovative solutions for the future.

## Figures and Tables

**Table 1:** High-Level Comparison of Melanin Synthesis.

Functional Category	Humans (Cutaneous and Neuromelanin)	Fungi (DOPA-Melanin and DHN-Melanin)
Precursor Molecules	Tyrosine, L-DOPA	Acetyl-CoA, Malonyl-CoA, or L-DOPA
Primary Enzymes	Tyrosinase (TYR), Tyrosinase-Related Protein 1 (TYRP1)	Polyketide Synthase (PKS), Laccase (LAC), Fungal Tyrosinases
Regulatory Control	MITF (Master Regulator), MC1R	Various Transcription Factors (e.g., MRR1), PKA signaling pathway
Primary Function	Pigmentation, UV protection, iron chelation (neuromelanin)	Virulence, protection from radiation, oxidative stress, and host defenses

**Table 2:** Detailed Comparison of Melanin in Humans and Fungi.

	Genes/Pathways	
	Humans	Fungi
Precursor Synthesis	Tyrosine Hydroxylase (TH): Synthesizes L-DOPA, a precursor for both neuromelanin and cutaneous melanin.	Acetyl-CoA/Malonyl-CoA Synthesis Pathways: Provide the building blocks for DHN-Melanin.
Melanin Synthesis Enzymes	Tyrosinase (TYR): The key rate-limiting enzyme for producing skin and hair melanin.	Polyketide Synthase (PKS): Essential for the DHN-melanin pathway (e.g., *ALB1* in *Aspergillus fumigatus*).
	Tyrosinase-Related Protein 1 (TYRP1) and Dopa-chrome Tautomerase (DCT): Further modify intermediates in the eumelanin (black/brown pigment) pathway.	Laccase (LAC): Key for the DOPA-melanin pathway (e.g., *LAC1* in *Cryptococcus neoformans*).
		Fungal Tyrosinases (TYR): Some fungi use tyrosinases, similar in function but different in origin from human TYR.
Regulation and Transport	MITF: The master transcription factor for melanocyte development and melanin gene expression.	Transcription Factors: Various factors control the expression of melanin genes in response to stress or developmental cues (e.g., *MRR1*).
	MC1R: A receptor that regulates the switch between producing black/brown eumelanin and red/yellow pheomelanin.	PKA Pathway: A signaling pathway that regulates melanin production in response to environmental signals.
	OCA2, SLC45A2: Transport proteins crucial for the proper function of melanosomes (the melanin-producing organelles).	
Stress Response and Adaptation	Oxidative Stress Response Genes (e.g., SOD2, HMOX1): Neuromelanin is believed to play a role in managing oxidative stress in neurons.	Stress Response Genes: Includes heat shock proteins and antioxidant enzymes that are often co-regulated with melanin.
	Inflammatory Response Genes: Related to the processes where neuromelanin is involved, such as in neurodegenerative diseases.	DNA Repair Genes: Melanin protects against DNA damage from UV radiation, and these genes work in concert.
		Virulence Factor Genes: Melanin is a key virulence factor in many pathogenic fungi, helping them survive host immune attacks.

**Table 3:** Location of Melanin by Cell Type and Species [90,91].

Kingdom	Organism Group	Example Species	Cell Types and Primary Location of Melanin
Animalia	Mammals	Humans, Mice	Melanocytes: Specialized cells in the epidermis (skin), hair follicles, and inner ear. They produce melanosomes and transfer them to surrounding keratinocytes.
			Retinal Pigment Epithelium (RPE) Cells: A layer of pigmented cells in the back of the eye, essential for vision.
			Uveal Melanocytes: Found in the iris and choroid of the eye.
			Neurons: Specific dopamine-producing neurons in the brain’s *substantia nigra* and *locus coeruleus* accumulate neuromelanin.
	Birds, Fish, Amphibians, Reptiles	Zebra Finch, Zebrafish, Frogs, Chameleons	Melanophores: Pigment-containing cells in the dermis. Unlike melanocytes, they can actively move pigment granules within the cell to cause rapid changes in skin color for camouflage or signaling. They are a type of chromatophore.
	Invertebrates	Fruit Flies, Squid, Octopus	Hemocytes (in insects): Immune cells that produce and deposit melanin around pathogens and wounds in a process called melanization.
			Epidermal Cells (in insects): Secrete melanin precursors into the cuticle, where they harden and darken the exoskeleton.
			Chromatophores (in cephalopods): Complex organs containing a sac of melanin pigment that can be rapidly expanded by muscles to change skin color.
Fungi	Yeasts, Molds, Mushrooms	*Cryptococcus neoformans*, *Aspergillus fumigatus*	Cell Wall: Melanin is commonly deposited directly into the fungal cell wall, making it a key structural and protective component of hyphae and yeast cells.
			Conidia (Spores): The outer layers of asexual spores are often heavily melanized, providing critical protection from UV radiation, heat, and enzymatic degradation.
			Sclerotia: Hardened, dormant masses of mycelium are melanized for long-term survival in harsh environments.
Plantae	Flowering Plants, Fruits	Sunflower, Banana, Date Palm	Seed Coat: Dark, melanin-like pigments (catechol melanins) are frequently deposited in the cells of the seed coat, offering protection.
			Damaged Tissues (Various Cell Types): The enzymatic browning of fruits and vegetables is a process of polymerization, creating melanin-like compounds. This occurs in various cells when they are damaged and exposed to oxygen.
			Bark and Heartwood: Cells in these tissues accumulate dark pigments that contribute to durability and defense against microbes.
Protista/Bacteria	Amoebas, Bacteria	*Acanthamoeba castellanii*, *Streptomyces* spp.	Extracellular Matrix/Secretion: Many bacteria synthesize and secrete pyomelanin or eumelanin into their surroundings, often as part of biofilm formation or for protection.
			Cell Wall/Spore Coat: Melanin can be integrated into the cell wall or the outer coat of cysts and endospores, enhancing resistance to environmental stressors.

## Data Availability

No new data were created or analyzed in this study.
